# Influence of peritoneal dialysis catheter type on dislocations and laxative use: a retrospective observational study

**DOI:** 10.1007/s40620-022-01329-6

**Published:** 2022-05-06

**Authors:** Gianmarco Sabiu, Marco Heidempergher, Cristina De Salvo, Maria Antonietta Orani, Chiara Tricella, Maurizio Gallieni

**Affiliations:** 1 Nephrology and Dialysis Unit, ASST Fatebenefratelli Sacco, Milano, Italy; 2 School of Nephrology, Università di Milano, Milano, Italy; 3 Department of Biomedical and Clinical Sciences, Università di Milano, Via Giovanni Battista Grassi 74, 20157 Milano, Italy

**Keywords:** Peritoneal dialysis, Peritoneal catheter, Laxative, Drug burden

## Abstract

**Background:**

There is currently no consensus regarding the optimal type of peritoneal dialysis (PD) catheter. Although few studies showed that weighted catheters result in lower complication rates and superior long-term outcomes than non-weighted catheters, there are no studies on the use of laxatives linked to catheter malfunction, a patient-related outcome potentially affecting the quality of life. Thus, we compared the burden of acute and chronic laxative use in a cohort of PD patients having either weighted or non-weighted catheters.

**Methods:**

We performed a single-center, retrospective, observational study in two renal units, comparing acute and chronic laxative therapy related to catheter drainage failure in a cohort of 74 PD patient,s divided by peritoneal dialysis catheter type. In addition, we evaluated the number of patients who experienced minor and major dislocations, catheter-related infection rate, hospitalization for catheter malfunctioning, episodes of catheter repositioning, and dropout from PD.

**Results:**

Laxative use was significantly more common among patients in the non-weighted catheter group (acute: 30.3% vs. 9.8%, p = 0.03; chronic: 36.4% vs. 12.2%; p≤0.02). Furthermore, weighted catheters were superior to non-weighted catheters for all the secondary outcomes (dislocations: 12.2% vs. 45.5%; p = 0.001).

**Conclusions:**

Weighted self-locating catheters have lower drainage failure, thus reducing the need and burden of acute and chronic laxative use among PD patients.

**Graphical abstract:**

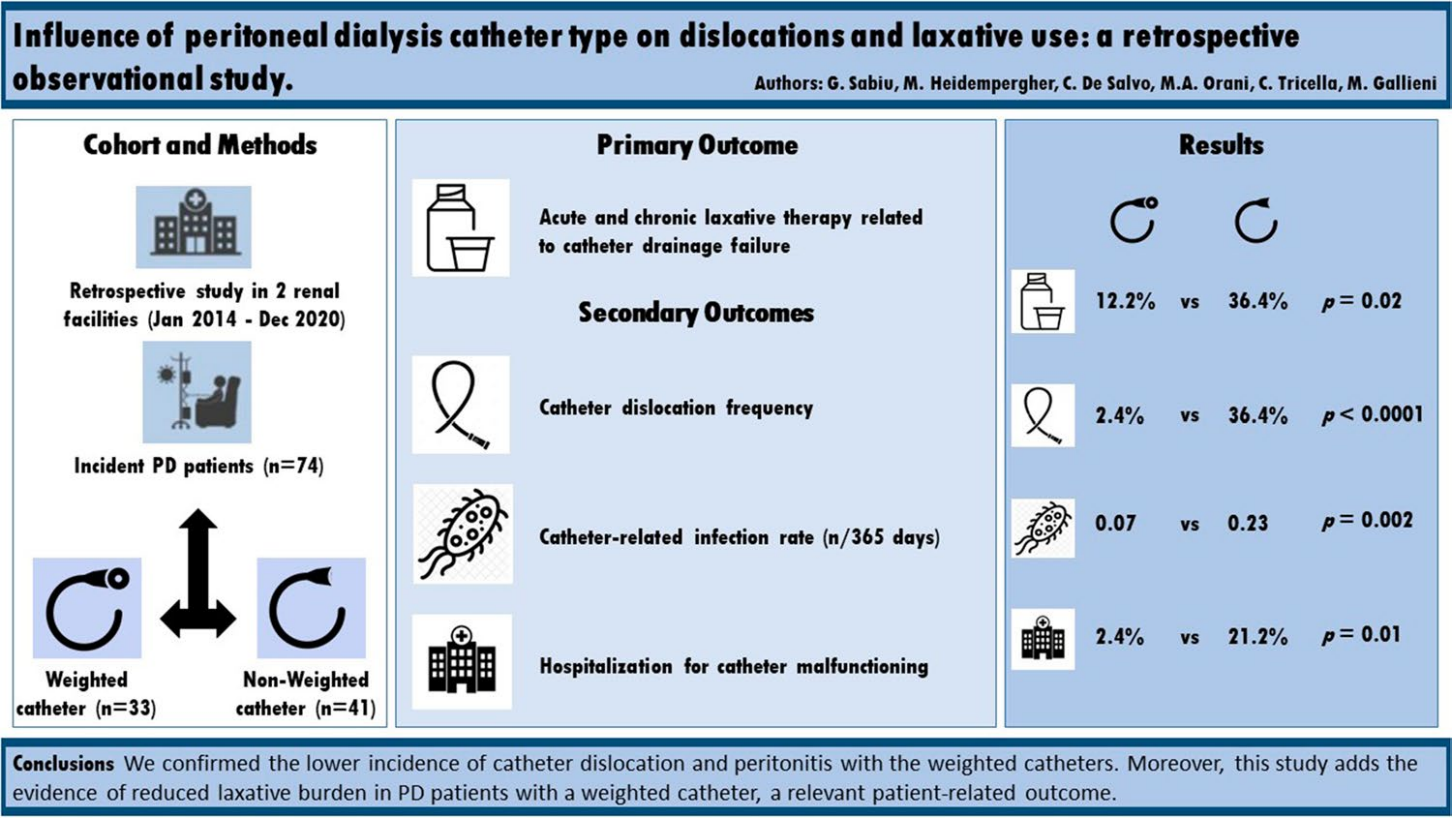

**Supplementary Information:**

The online version contains supplementary material available at 10.1007/s40620-022-01329-6.

## Introduction

It is established that well-functioning catheters are associated with a lower incidence of peritonitis and better efficiency of dialysis, thereby representing an essential tool to guarantee optimal Peritoneal Dialysis (PD) delivery. However, currently there is no consensus regarding the best catheter to perform PD [1].

Ideally, well-functioning catheters should ensure a good quality of treatment, thus significantly reducing mechanical complications. Indeed, mechanical complications represent one of the leading causes of dropout among PD patients, accounting for almost 40% of PD to hemodialysis shifts in the first three months and almost 25% dropout overall [2].

There are various types of available PD catheters. They differ with regard to design, including the number of cuffs, shape of the subcutaneous tract (straight vs. swan neck), and of the intra-peritoneal tract (straight vs. coiled) [3]. Moreover, there is a particular category of PD catheters known as "self-locating catheters" designed by Di Paolo in 1992. Self-locating catheters, also known as weighted catheters, are provided with a 12 gr weighted tungsten tip at the end of a classic Tenckhoff catheter to avoid catheter migration [4].

Some observational studies[5, 6], and some small randomized controlled trials (RCTs) [7, 8], showed beneficial effects on mechanical complications with self-locating catheters. According to these studies, self-locating catheters are less prone to tip migration, resulting in less drainage failure events and eventually reducing catheter-related infections. However, despite these promising findings, self-locating catheters usage is limited [9], and an adequately powered RCT is still  lacking.

While awaiting more robust evidence, acute and chronic laxative use has become widespread among PD patients. Indeed, laxatives induce peristalsis that prevents tip migration [10]. Although helpful in preventing catheter migration, chronic laxative use can easily turn into laxative abuse in this subgroup of patients, affected by chronic constipation and frequent mechanical complications. Laxative abuse could lead to electrolyte and acid/base changes, patient discomfort, abdominal cramps, and dehydration due to factitious diarrhea [11]. In addition, laxative use may provoke transmural migration of enteral microflora to the peritoneal cavity, predisposing to peritonitis [12].

Our study aimed to compare the mechanical complications related to PD catheter type as well as burden of laxative use in a cohort of PD patients with either weighted or non-weighted catheters.

## Methods

We conducted a single-center, retrospective, observational study among all the incident PD patients followed-up in two Renal Units of the same institution in Milan, Italy, from 2014 until 2020.

We included all patients above 18 years of age and on dialysis for at least three months, excluding those with shorter follow-up to avoid confounding factors such as early mechanical complications and unknown adhesion syndrome.

We divided patients into two cohorts based on catheter type: weighted, with self-locating properties (Care-Cath® B.Braun Avitum, Mirandola, Italy) and non-weighted, standard straight Tenckhoff catheters. Weighted catheters progressively replaced the Tenckhoff catheters and were widely adopted in the two units in 2019.

We collected data on catheter tip migration (highlighted by abdominal x-ray), acute and chronic laxative use, PD dropout linked to catheter malfunction, and catheter-related infections (peritonitis and exit-site infections).

The primary endpoint of the study was to evaluate laxative use in PD patients to either prevent, or treat drainage failure.

Secondary outcomes included episodes of dislocations, HD shift due to catheter malfunction (PD dropout rate), hospitalization for malfunctioning, catheter repositioning, catheter-related infections, and cuff-shavings.

Chronic laxative therapy – for which a definition is lacking – was reported in patients who chronically used laxatives at least three times a week. Chronic laxative use in otherwise non-constipated patients was considered a parameter of catheter malfunctioning. Patients on chronic laxative therapy for constipation were excluded from the final analysis.

We evaluated the percentage of patients that experienced drainage failure and needed acute laxative use, and the percentage of patients with at least one abdominal x-ray with evidence of tip migration. We distinguished between minor dislocations, for which acute laxative use was followed by complete resolution, and major dislocations, highlighted through catheter tip migration seen by abdominal x-ray after acute laxative use. We excluded patients already on treatment with chronic laxatives from the acute laxative use group.

PD dropout is expressed as the percentage of patients who had to change renal replacement therapy, shifting to hemodialysis (HD), because of PD catheter malfunctioning. Episodes of hospitalization and catheter repositioning were reported as number of patients hospitalized due to catheter-related mechanical issues*.*

Finally, catheter-related infections were measured following the 2017 International Society of Peritoneal Dialysis (ISPD) guidelines [13].

The chi-square test, the T-test, and the Mann Whitney U test were used for the statistical analysis of baseline data and of primary and secondary endpoints. The mid-p exact test evaluated the difference between infection rates, while multiple logistic regression was used to analyze the correlation between independent variables.

The Medical Ethics Committee of the “ASST-Fatebenefratelli-Sacco” approved the protocol. All patients provided written informed consent to participate in the study. The authors observed the Helsinki guidelines.

## Results

**Fig. 1:**
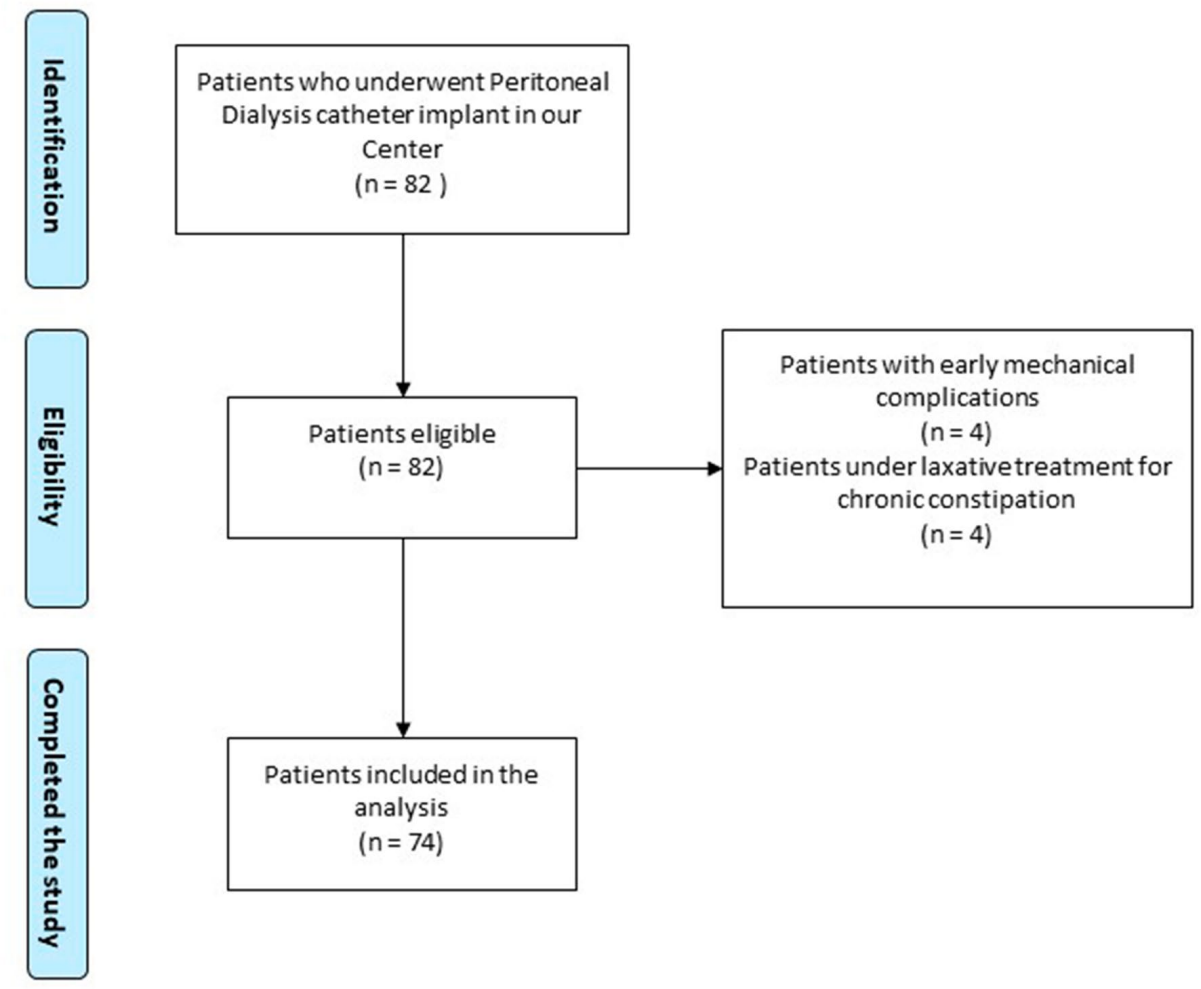
Patients selection and eligibility

We identified 82 eligible patients and excluded 8 of them, 4 due to early mechanical complications and 4 to chronic constipation, respectively (Fig.1). Of note, 2 of the 4 patients who were excluded because of chronic constipation had a non-weighted catheter. Finally, 41 and 33 patients were included in the non-weighted catheter and weighted catheter groups, respectively.

The two study groups were balanced with regard to age, BMI, prevalence of diabetes, diverticulosis and percentage of patients who had undergone previous abdominal surgery. Major differences between the two populations are the widespread use of automated peritoneal dialysis (APD) in the weighted catheter group (80.5% vs 60.6%; p = 0.07), and the older catheters in the non-weighted catheter group (median: 851 days vs 516 days; p = 0.07), reflecting the more recent introduction of the weighted catheter in the two PD units. Furthermore, there was a non-significant difference in type of laxative used between the two groups, with a higher percentage of patients using lactulose rather than Movicol® in the weighted catheter group compared to the non-weighted catheter group (80% vs 41.7%; p = 0.44). All the catheters were implanted with the mini-laparotomy approach.

Baseline characteristics of the study population are provided in Table 1.

**Table 1:** Baseline characteristics of the patients

	Non-weighted catheter (n = 33)	Weighted catheter (n = 41)	*p* value
Age, *mean* ± *SD*	69.7 ± 14.1	70.6 ± 14	NS
Sex: male, *n. (%)*	20/33 (60.6)	28/41 (68.3)	NS
Sex: female, *n. (%)*	13/33 (39.4)	13/35 (31.7)	NS
BMI (kg/m2), *mean* ± *SD*	23.5 ± 3.7	23.4 ± 3.1	NS
Diabetes	17/33 (51.5)	17/41 (41.5)	NS
Diverticulosis	4/33 (12.1)	5/41 (12.2)	NS
Catheter vintage (days), *median (IQR)*	851 (518–1500)	516 (296–1235)	NS (0.07)
CAPD - APD, *%*	39.4 – 60.6	19.5 – 80.5	NS (0.07)
Previous abdominal surgery, *n. (%)*	9/33 (27.3)	11/41 (26.8)	NS

*APD* automated peritoneal dialysis, *BMI* body mass index, *CAPD* continuous ambulatory peritoneal dialysis

Regarding the primary endpoint, acute and chronic laxative use was more common among non-weighted catheter patients: 30.3 % vs 9.8% (*p* = 0.03) and 36.4% vs 12.2% (*p* = 0.02), respectively.

Dislocations were also more frequent among patients with a non-weighted catheter, with a significant difference in radiologically-proven catheter tip migration: 36.4% vs 2.4% (*p* < 0.0001). The number of patients who experienced either a clinically diagnosed dislocation, or a radiologically-proven catheter tip migration, was much higher among the non-weighted catheter group (45.5% vs 12.2%; *p* = 0.001).

Multiple logistic regression analysis showed that among the analyzed independent variables (Table 2), only the type of catheter was significantly related to the primary outcome (OR 4.22; CI 95% 1.349 to 15.09; p = 0.018).

**Table 2:** Multiple logistic regression analysis – Predictors of laxative use for catheter malfunctioning in peritoneal dialysis patients

	Odds Ratio	95% CI	*p* value
Non-weighted catheter	4.22	1.349–15.09	0.0175
Previous abdominal surgery	1.60	0.433–5.601	NS (0.47)
Diverticulosis	1.69	0.289–8.485	NS (0.53)

Catheter-related infections also differed between the two groups, with a significant reduction in peritonitis incidence among patients in the weighted catheter group: 0.07/365 days vs 0.26/365 days (*p* = 0.002). The incidence rate ratio (IRR) of peritonitis was 0.27 (73% reduction), whereas no differences were observed regarding exit-site infections.

Hospitalization and catheter repositioning were also significantly more frequent among patients in the non-weighted group, 21.2% vs. 2.4% (*p* = 0.01).

The dropout rate for mechanical complications was higher among the non-weighted catheter group, even if the number of events was too small to carry out a statistically reliable comparison (9% vs 0%; *p* = NS). Detailed results are provided in Table 3.

**Table 3:** Primary and secondary endpoints

	Weighted catheter (n = 41)	Non-weighted catheter (n = 33)	*p*
Chronic laxative use to prevent drainage failure *N. of patients (%)*	5 (12.2)	12 (36.4)	0.02
Minor dislocations (Acute laxative use) *N. of patients (%)*	4 (9.8)	10 (30.3)	0.03
Major dislocations (Catheter tip migration at x-ray) *N. of patients (%)*	1 (2.4)	12 (36.4)	< 0.0001
Total dislocations *N. of patients (%)*	5 (12.2)	15 (45.5)	0.001
Peritoneal Dialysis dropout for mechanical complications *N. of patients (%)*	0 (0)	3 (9.1)	NS
Hospitalization for catheter malfunctioning *N. of patients (%)*	1 (2.4)	7 (21.2)	0.01
Catheter repositioning *N. of patients (%)*	1 (2.4)	7 (21.2)	0.01
Peritonitis *N. episodes/year (95% CI)*	0.07 (0.03–0.15)	0.26 (0.17–0.39)	0.002
Exit-site infections *N. episodes/year (95% CI)*	0.15 (0.09–0.26)	0.17 (0.1–0.27)	NS
Cuff-shaving *N. of patients (%)*	5 (12.2)	6 (18.2)	NS

## Discussion

In this single-center, retrospective, observational study, we compared the frequency of mechanical complications between two types of peritoneal catheters, as well as their infection rates. Our results show that weighted catheters were associated with reduced rates of mechanical complications (such as catheter dislocations), peritonitis, and hospitalization for catheter malfunctioning and repositioning. These findings are consistent with previously reported studies [5–8].

Furthermore, we analyzed two new parameters: acute and chronic laxative use. We report the widespread use of laxatives in otherwise non-constipated patients within the non-weighted catheter group. To our knowledge, this is the first study to evaluate these parameters in a comparison between two PD catheter types.

Catheter tip migration is a common complication with non-weighted catheters, with an incidence as high as 24% [14]. When migration occurs, catheter functioning is affected, leading to drainage failure [15]. Restoration of the proper catheter position can be achieved through non-invasive or minimally invasive techniques, such as laxative use or repositioning with a metal wire, respectively. However, refractory cases often require surgical revision or removal and replacement of the catheter [16]. On the other hand, weighted catheter dislocation can often be reversed more easily and non-invasively by positional changes under radioscopic control.

Avoiding catheter tip migration should be one of the main goals in PD. Indeed, in our cohorts it reduced hospitalization rate, laxative use, and catheter manipulation.

**Fig. 2:**
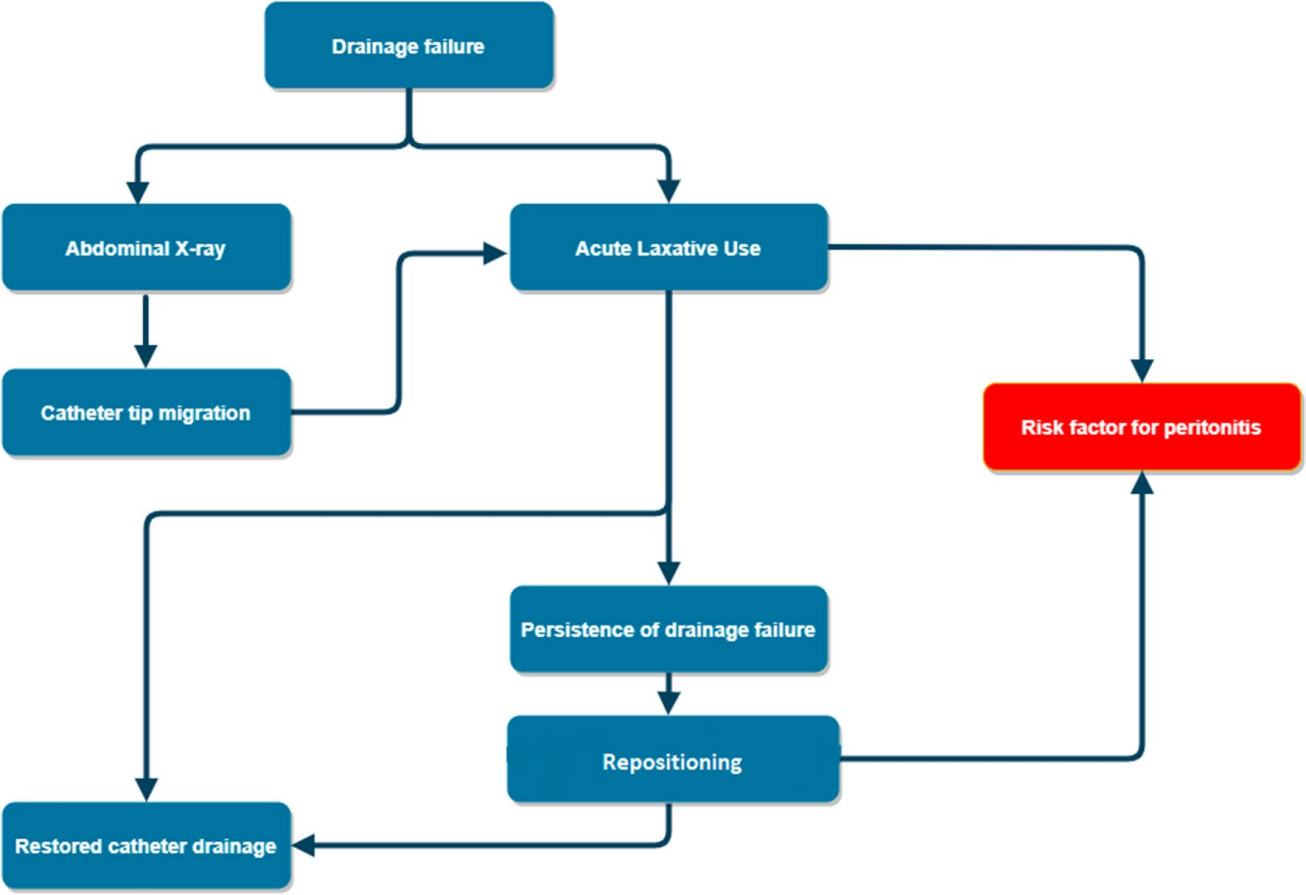
Drainage failure management flowchart. Catheter repositioning, as well as the acute laxative use, increase the risk of catheter-related infections

Patients on PD are prone to chronic constipation because of aging, hypothyroidism, hypercalcemia, diabetes, and autonomic nervous system dysfunction. Moreover, most PD patients receive treatments that can potentiate constipation, such as phosphate and potassium binders, calcium channel blockers, opioids, and iron preparations [17, 18]. Constipation in patients receiving PD is associated with increased risk of mechanical and infectious complications, thus affecting catheter drainage and promoting laxative use [19]. However, treatment of constipation with laxatives may predispose to bacterial translocation and peritonitis in PD patients (Fig.2) [12, 17].

In our study, the weighted catheter reduced laxative use, both in the acute and chronic settings. Moreover, laxative use was an indirect index to determine the drainage failure rate and appeared to be related to the peritonitis rate, the risk of which was reduced in the weighted catheter group (IRR 0.27).

The consistency of both the primary and the secondary outcomes represents the major strength of this study. These findings prompt a correlation between the number of dislocations, laxative use, and peritonitis rate. Reducing the incidence of dislocations guarantees optimal peritoneal dialysis quality and places patients at a lower incidence of infections depending on catheter manipulation and laxative use (Fig1).

The recent ISPD practice recommendations on the prescription of high-quality, goal-directed PD [20] underscored the concept that the well-being of the person on PD involves much more than just the removal of toxins. Rather, the healthcare system should focus on the person undergoing PD, beyond the medical perspective of the “patient” status, with goal-directed dialysis delivery. Avoiding or at least limiting the use of laxatives and their consequences should be a goal of PD care that can be achieved by adopting weighted, self-locating catheters.

There are some limitations in this study. Firstly, the retrospective nature of the study results in some biases, such as selection and information bias. Although they represent the whole of incident patients in our center, the small number of patients included could open the study to a certain degree of variability. However, the two groups were well balanced, except for PD technique and catheter vintage, which presumably are not factors affecting the dislocation rate or laxative use.

A recent systematic review and meta-analysis suggested that weighted catheters result in lower complication rates and superior long-term outcomes compared to non-weighted catheters [21]. This study adds observational evidence of the weighted catheter benefits. A randomized controlled trial should confirm the superiority of the weighted catheter over the non-weighted catheter.

## Conclusions

We confirmed the lower incidence of catheter dislocation with the weighted catheters. What this study adds is the evidence of reduced laxative burden in PD patients with a weighted catheter, a relevant patient-related outcome, which in addition seems to be related to a reduction in peritonitis rate.

Our study is another proof-of-concept suggesting the need for a well-designed, sufficiently powered, large study to compare the two types of catheters.

## Supplementary Information

Below is the link to the electronic supplementary material.

Supplementary file1 (XLS 60 KB)
